# Four novel *Acinetobacter lwoffii* strains isolated from the milk of cows in China with subclinical mastitis

**DOI:** 10.1186/s12917-024-04119-3

**Published:** 2024-06-26

**Authors:** Qiang Chen, Wensi Zhou, Yuening Cheng, Guisheng Wang, Zhihao San, Li Guo, Liming Liu, Cuiqing Zhao, Na Sun

**Affiliations:** 1 College of Animal Science and Technology, Jilin Agricultural Science and Technology University, Jilin, China; 2 Key Laboratory of Special Animal Epidemic Disease, Ministry of Agriculture, Institute of Special Economic Animals and Plants, Chinese Academy of Agricultural Sciences, Changchun, China; 3 Shandong Provincial Center for Animal Disease Control and Prevention, Jinan, China

**Keywords:** *Acinetobacter lwoffii*, Milk, Bovine, Subclinical mastitis, Resistance genes

## Abstract

**Background:**

*Acinetobacter lwoffii* (*A. lwoffii*) is a Gram-negative bacteria common in the environment, and it is the normal flora in human respiratory and digestive tracts. The bacteria is a zoonotic and opportunistic pathogen that causes various infections, including nosocomial infections. The aim of this study was to identify *A. lwoffii* strains isolated from bovine milk with subclinical mastitis in China and get a better understanding of its antimicrobial susceptibility and resistance profile. This is the first study to analyze the drug resistance spectrum and corresponding mechanisms of *A. lwoffii* isolated in raw milk.

**Results:**

Four *A. lwoffii* strains were isolated by PCR method. Genetic evolution analysis using the neighbor-joining method showed that the four strains had a high homology with *Acinetobacter lwoffii*. The strains were resistant to several antibiotics and carried 17 drug-resistance genes across them. Specifically, among 23 antibiotics, the strains were completely susceptible to 6 antibiotics, including doxycycline, erythromycin, polymyxin, clindamycin, imipenem, and meropenem. In addition, the strains showed variable resistance patterns. A total of 17 resistance genes, including plasmid-mediated resistance genes, were detected across the four strains. These genes mediated resistance to 5 classes of antimicrobials, including beta-lactam, aminoglycosides, fluoroquinolones, tetracycline, sulfonamides, and chloramphenicol.

**Conclusion:**

These findings indicated that multi-drug resistant *Acinetobacter lwoffii* strains exist in raw milk of bovine with subclinical mastitis. *Acinetobacter lwoffii* are widespread in natural environmental samples, including water, soil, bathtub, soap box, skin, pharynx, conjunctiva, saliva, gastrointestinal tract, and vaginal secretions. The strains carry resistance genes in mobile genetic elements to enhance the spread of these genes. Therefore, more attention should be paid to epidemiological surveillance and drug resistant *A. lwoffii*.

## Background

Mastitis is a common disease in dairy cows, which threatens the development of the dairy cattle industry worldwide. The disease causes significant economic losses by reducing milk production and milk quality [1, 2]. Mastitis is caused by many pathogens, and the predisposing factors include a dirty environment, improper feeding and management, hormone disorders, breast defects, and other factors. Bacteria, viruses, and fungi are the main causes of mastitis. The causal microorganisms of mastitis are complex. In general, *Staphylococcus*, *Streptococcus*, and *Escherichia coli* are the main pathogenic bacteria that cause mastitis, followed by *Corynebacterium pyogenes*, *Pseudomonas aeruginosa, Pasteurella* and *Klebsiella*. There are two types of mastitis based on clinical manifestations: clinical mastitis (CM) and subclinical mastitis (SCM). Subclinical mastitis is more prevalent than clinical mastitis, and cow-to-cow transmission is the primary route through which the disease spreads [3]. Subclinical mastitis has a systemic effect on the reproduction capacity of the infected animal [4], and it can be diagnosed based on the presence of inflammatory mediators and specific bacteria in milk and a reduction in milk production [5].

*Streptococcus* and *Staphylococcus* are the main bacteria species that cause subclinical mastitis. Recent studies have shown that several bacterial species associated with bovine mastitis, such as environmentally ubiquitous *Acinetobacter*, *Bacillus*, *Enterobacter*, and *Enterococcus*, are readily isolated from milk samples [6, 7]. These pathogens are developing multiple drug resistance (MDR) to common antimicrobial agents used in mastitis therapy. *Acinetobacter* are Gram-negative bacillus with 112 Acinetobacter species in this genus. The majority of species are nonpathogenic types readily available in the environmental materials. Members of the *Acinetobacter* genus easily cause infection in immunocompromised individuals and animals. The most common infections are nosocomial, predominantly respiratory tract infections, septicemia, meningitis, endocarditis, wound and skin infections, and urogenital tract infections. The most common *Acinetobacter* species that cause infections is *Acinetobacter baumannii*, followed by *Acinetobacter calcoaceticus* and *Acinetobacter lwoffii* [8]. All species are ubiquitous in nature and can easily be isolated from soil, water, food, and sewage [9]. In this study, we isolated 4 *A. lwoffii* strains from raw milk samples of cows with subclinical mastitis in Jilin Province in China in 2021. The antimicrobial susceptibility, resistance, and the genes conferring the resistance in the isolates were determined.

## Results

### Identification of strains and genetic evolution analysis

The milk samples from cows with subclinical mastitis were analyzed for the presence of mastitis-related bacteria. Four *Acinetobacter* strains, including JL1, JL2, JL3, and JL4, were obtained from the analyzed raw milk samples.

**Fig. 1:**
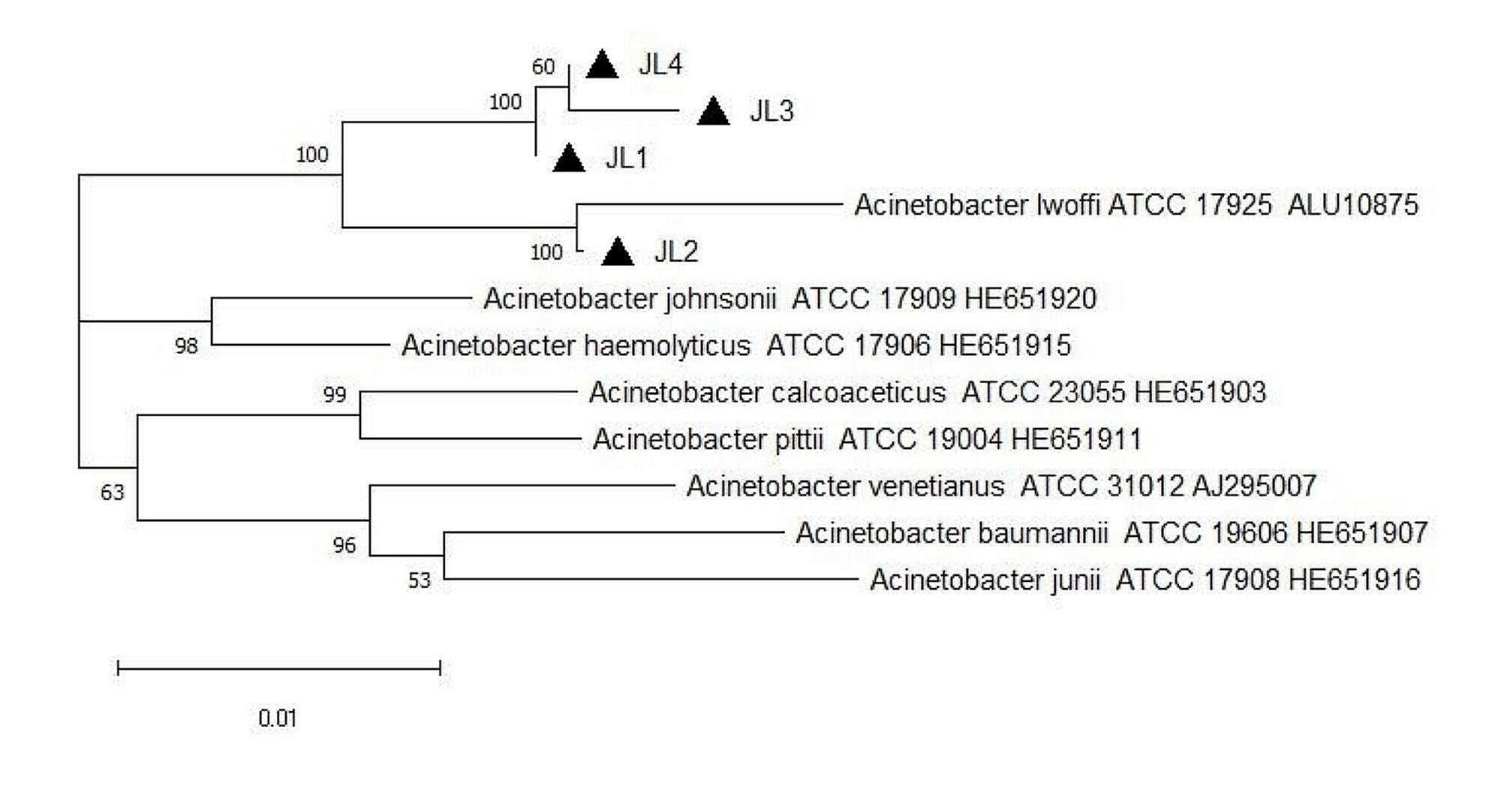
Phylogenetic relationship of 16s rRNA gene between milk *Acinetobacter* strains and selected reference strains. The tree was constructed using Neighbor-Joining method implemented in MEGA7.0 software with p-distances and 1,000 bootstrap replicates. The samples are labeled with black triangle

A phylogenetic tree constructed using the neighbor-joining method showed that the four strains grouped together in one clade and showed high homology with *Acinetobacter lwoffii* (Fig. 1).

### Antimicrobial susceptibility phenotypes

**Fig. 2:**
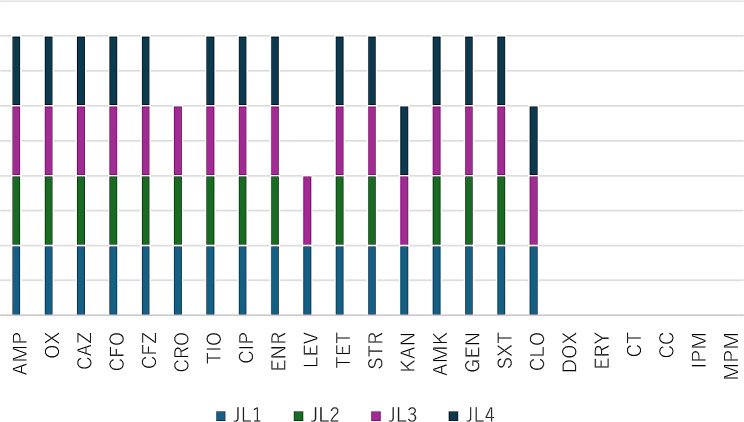
The results of multidrug resistance in isolates Note: AMP: Ampicillin; OX: Oxacillin; CAZ: Ceftazidime; CFO: Cefoxitin; CFZ: Cefazolin; CRO: Ceftriaxone; TIO: Ceftiofur; CIP: Ciprofloxacin; ENR: Enrofloxacin; LEV: Levofloxacin; TET: Tetracycline; STR: Streptomycin; KAN: Kanamycin; AMK: Amikacin; GEN: Gentamicin; SXT: Trimethoprim/sulfamethoxazole; CLO: Chloramphenicol; DOX: Doxycycline; ERY: Erythromycin; CT: Polymyxin; CC: Clindamycin; IPM: Imipenem; MPM: Meropenem

The susceptibility and resistance profile of the four strains were tested against 23 antibiotics. The antimicrobial susceptibility results showed that the four strains were resistant to multiple drugs (Fig. 2). All the strains were resistant to ampicillin, oxacillin, ceftazidime, cefoxitin, cefazolin, ceftiofur, ciprofloxacin, enrofloxacin, tetracycline, amikacin, streptomycin, gentamicin, and trimethoprim/sulfamethoxazole (JL1 and JL3 isolates were resistant to Ampicillin-Oxacillin-Ceftazidime-.

Cefoxitin-Cefazolin-Ceftriaxone-Ceftiofur-Ciprofloxacin-Enrofloxacin-Levofloxacin-Tetracycline-Streptomycin-Kanamycin-Amikacin-Gentamicin-Chloramphenicol-Trimethoprim/sulfamethoxazole, JL2 isolate was resistant to Ampicillin-Oxacillin-Ceftazime-Cefoxitin-Cefazolin-Ceftriax.

one-Ceftiofur-Ciprofloxacin-Enrofloxacin-Tetracycline-Streptomycin-Amikacin-Gentamicin-Trimethoprim/sulfamethoxazole, and JL4 isolate was resistant to Ampicillin-Oxacillin-Cefoxitin-Ceftazidime-Cefazolin-C.

eftiofur-Ciprofloxacin-Enrofloxacin-Tetracycline-Streptomycin-Kanamycin-Amikacin-Gentamicin-Chloramphenicol-Trimethoprim/sulfamethoxazole). However, they were more susceptible to doxycycline, erythromycin, polymyxin, clindamycin, imipenem and meropenem. Notably, the strains displayed multi-drug resistance with variable patterns.

### Presence of resistance genes

The distribution of resistance genes across the four strains is shown in Table 1. Two beta-lactamase genes were detected. *Bla*_*TEM*_ was detected in all four strains, while *bla*_*SHV*_ was detected in two isolates. Among the aminoglycoside resistance genes, 4 aminoglycoside modifying enzyme genes were identified. The *aadA1* gene which confers resistance to streptomycin was detected in all 4 isolates. The *aac(3’)-IIc* gene, which confers resistance to gentamicin, was detected in all 4 isolates. The *aph(3’)-VII* gene, which causes resistance to kanamycin, was detected in all 4 isolates. The *aac(6’)-Ib* gene, which causes resistance to kanamycin and amikacin, was detected in all 4 isolates. Only one 16 S rRNA methylase gene *rmtB* was detected in JL1. Three plasmid-based antibiotic resistance-associated genes were detected. *OqxA* was present in all four, *oqxB* was also present in all four, and *qnrB* was present in two isolates. Among the tetracycline and sulfonamide resistance genes, *tet(A)*, *tet(C)*, and *tet(G)* were present in four isolates, *tet(K)* was present in one, *sul1* was present in two, while *sul2* was present in three isolates. Among the chloramphenicol resistance genes, the *cat2* gene was detected in all 4 isolates.

**Table 1:** The results of resistance genes in isolates

Isolates	Resistance genes	
JL1	Beta-lactam genes	*bla*_TEM_, *bla*_SHV_
	Aminoglycosides	*aadA1*, *aph(3’)-VII*, *aac(3’)-IIc*, *aac(6’)-Ib*, *rmtB*
	Fluoroquinolones	*qnrB*, *oqxA*, *oqxB*
	Tetracycline	*tet(A)*, *tet(G)*, *tet(C)*
	Sulfonamides	*sul1*
	Chloramphenicol	*cat2*
JL2	Beta-lactam genes	*bla*_TEM_
	Aminoglycosides	*aadA1*, *aph(3’)-VII*, *aac(3’)-IIc*, *aac(6’)-Ib*
	Fluoroquinolones	*oqxA*, *oqxB*
	Tetracycline	*tet(A)*, *tet(G)*, *tet(C)*
	Sulfonamides	*sul1,sul2*
	Chloramphenicol	*cat2*
JL3	Beta-lactam genes	*bla*_TEM_, *bla*_SHV_
	Aminoglycosides	*aadA1*, *aph(3’)-VII*, *aac(3’)-IIc*, *aac(6’)-Ib*
	Fluoroquinolones	*oqxA*, *oqxB*
	Tetracycline	*tet(A)*, *tet(G)*, *tet(C)*
	Sulfonamides	*sul2*
	Chloramphenicol	*cat2*
JL4	Beta-lactam genes	*bla*_TEM_
	Aminoglycosides	*aadA1*, *aph(3’)-VII*, *aac(3’)-IIc*, *aac(6’)-Ib*
	Fluoroquinolones	*qnrB*, *oqxA*, *oqxB*
	Tetracycline	*tet(A)*, *tet(G)*, *tet(C)*, *tet(K)*
	Sulfonamides	*sul2*
	Chloramphenicol	*cat2*

## Discussion

Mastitis is a common disease in dairy cows, which influence the lactation period and milk production. The quality and shelf life of raw milk and related products will be reduced by the increase of microbiology [10]. Pathogenic bacteria in raw milk could cause serious food safety and even affect human health. Because mastitis is debilitating and painfulit, also touches on animal welfare issues [11]. *Staphylococcus*, *Pseudomonas*, *Streptococcus*, *Pasteurella*, *Enterobacter*, *Klebsiella*, *Corynebacterium*, *Enhydrobacter*, *Bacillus*, *Lactococcus*, *Lactobacillus*, *Paenibacillus*, *Bacteroides*, *Massilia*, *Chryseobacterium*, *Enterococcus*, *Psychrobacter* and *Acinetobacter* have been previously detected in raw milk of cows with mastitis [12–19]. In this study, 4 *Acinetobacter lwoffii* strains were detected in raw milk. This study is very important because little is known about the role of foods in the transmission of *Acinetobacter spp.* No standard protocols for recovering these species from foods exist [20, 21]. *Acinetobacter lwoffii* is an aerobic, Gram-negative coccobacillus common in the environment and a normal flora in the human respiratory and digestive tract [22]. According to the most recent scientific literature, members of the *Acinetobacter* genus are the second most common nonfermenting pathogens isolated from clinical samples after *Pseudomonas aeruginosa* [23]. *Acinetobacter* strains also colonize animals respiratory and urinary tract, including food animals, fish, chickens, birds, and dogs [24–28]. *Acinetobacter* is widely distributed in the external environment, such as water, soil, baths, soap boxes and other wet places [29, 30]. The bacterium has strong adhesion and easily adheres to various medical materials, where it may become a storage source of bacterial infections. These bacteria survive on inanimate objects, in dry conditions, in dust, and in moist conditions for several days. A study showed that in raw milk of cows with mastitis, the detection rate of *Acinetobacter baumannii* was higher than *Acinetobacter lwoffii* [31]. However, *Acinetobacter baumannii* is still the most common *Acinetobacter* species that causes infections. Other prominent species include *Acinetobacter ursingii*, *Acinetobacter parvus* and among all, *Acinetobacter lwoffii* has been increasingly reported. Ribeiro Júnior JC isolated 9 *Acinetobacter lwoffii* strains from 20 refrigerated raw milk samples [32].

Since bovine mastitis results in huge economic losses, its prevention and treatment have attracted global attention. Antibiotics are the main treatment options for the disease. In recent years, *Acinetobacter* species have been the most common pathogens associated with opportunistic infections resistant to multiple antibiotic classes. In this study, the *A. lwoffii* strains isolated were resistant to multiple antibiotics at variable patterns. All the strains were only susceptible to 6 out of the 23 commonly used antibiotics. In a previous study, the *Acinetobacter* strains detected from milk samples of cows suffering from clinical mastitis were resistant to all the antibiotics tested (oxytetracycline, vancomycin, lincomycin, nitrofurantoin, ceftriaxone-tazobactam, cefotaxime, erythromycin, amoxicillin-sulbactam, and penicillin) [6]. *Acinetobacter* species isolated by Raylson Pereira de Oliveira showed variable phenotypic resistance to antimicrobials and were completely resistant to ampicillin, penicillin, and vancomycin [31]. *Acinetobacter* strains which isolated from human milk were resistant to oxacillin, ampicillin, clindamycin, cephalothin, amoxicillin and erythromycin [33]. *Acinetobacter* species isolated from birds on a free-range farm were resistant to ampicillin, cefazolin, ceftazidime, chloramphenicol, nitrofurantoin, rifampicin and tetracycline which are on the WHO list of essential medicines [34]. The resistance profile of the strains detected in this study has never been reported in the past. The difference in the drug resistance pattern for *A. lwoffii* is due to the use of different drugs in different regions. The high antimicrobial resistance of *A. lwoffii* strains may be due to the overuse and abuse of antimicrobials in disease treatment. *Acinetobacter baumannii* as the important pathogen in healthcare associated infections shows serious multiple-drug resistance [35, 36]. There are not any *Acinetobacter baumannii* strain isolated from the analyzed milk samples in this study. However, it suggested that other *Acinetobacter* species isolates may play a role in maintaining severe antibiotic resistance in milk.

There are many reports concerning the resistance mechanism of *Acinetobacter lwoffii* have been published. In Sofia Mindlin’s study, *Acinetobacter lwoffii* carried antibiotic-resistant genes (heavy metal resistance) in plasmids [37]. Liang detected a novel plasmid-encoded ANT(3”)-IId in *Acinetobacter lwoffi* strain isolated from a chick on an animal farm in China [38]. Two β-lactamase-encoding genes, OXA-496 and OXA-537, were for the first time reported in *Acinetobacter lwoffii* and *Acinetobacter schindleri* isolates from a chicken farm [39]. In this study, a total of 17 genes that mediate resistance to beta-lactam, aminoglycosides, fluoroquinolones, tetracycline, sulfonamides, and chloramphenicol were detected across the four strains isolated, and some of these genes were carried in the plasmid. In this study, the relationship between the carrying of resistance determinants and the phenotypic resistance profile were not coincident. Some strains having resistance genes while susceptible to the antimicrobial. Maybe the resistance genes were not expressed, so that they did not show resistance to the antimicrobial. Some strains were resistant to antimicrobial without the related resistance genes. It was possible that there were other resistance mechanism. The metabolic abilities of *Acinetobacter spp.* are often attributed to their plasmid-encoded genes because these genes encode proteins that can degrade organic compounds [40]. Plasmid mediated gene transfer plays an important role in the transmission of antibiotic resistance genes, pathogen degradation pathways and pathogenicity determinants. The transfer of mobile genetic elements such as plasmids, insertion sequences (ISs), transposons and integrons play an important role in the acquisition of resistance determinants or features providing a selective advantage [41]. These transposable elements can move within the bacterial genome. Plasmid-based genes encode numerous features that provide a selective advantage to the bacteria, and they can be transferred horizontally to other bacteria of the same or different species. Therefore, plasmids are believed to play an essential role in the evolutionary events of a given microbial community [42]. Numerous articles have documented the presence of *Acinetobacter spp.* in raw milk, dairy products, and powdered bovine milk [21]. *Acinetobacter spp.* are common microbes found throughout nature. *Acinetobacter spp.* in raw milk may have originated from environmental sources. Also, *Acinetobacter spp.* can contaminate other animals, people, medical devices, and environmental factors. In addition, *Acinetobacter spp.* strains that carry plasmid-based resistance genes may transfer these genes to other strains through the horizontal mechanism. Acquisition of plasmids that mediate antibiotic resistance transfers these traits to the recipient bacteria, which seriously threatens effective clinical treatment of diseases caused by such bacteria. The prevalence and mechanisms of antibiotic resistance have been widely reported, but the transfer mechanisms of multi-drug resistant genes are remain unclear. The research of antibiotic resistance gene transfer were good for devising innovative solutions to combat the current antibiotic resistance crisis [43].

The prevalence and mechanisms associated with antibiotic resistance have been widely reported, but the mechanisms of multidrug resistance gene transfer. It is important to continue antibiotic resistance gene transfer to design innovative solutions to combat the current antibiotic resistance crisis.

## Conclusion

We isolated 4 *A. lwoffii* stains from raw milk samples of cows with subclinical mastitis in China. Genetic evolution analysis with the neighbor-joining method showed that the 4 strains displayed a high homology with *Acinetobacter lwoffii*. The antimicrobial susceptibility test which using Kirby-Bauer disk diffusion and refered to Clinical and Laboratory Standards Institute results showed that all the strains were multi-drug resistant and were only completely susceptible to 6 of the 23 tested antibiotics. *Acinetobacter lwoffii* strains, which inhabit the udder of cows, showed considerably variable multi-drug resistance patterns. The antibiotic resistance genes were diverse and varied across the four strains. A total of 17 resistance-associated genes, including plasmid-based genes, were detected. These genes promoted resistance against 5 drug categories, including beta-lactam, aminoglycosides, fluoroquinolones, tetracycline, sulfonamides, and chloramphenicol. Our findings suggest that *Acinetobacter lwoffii* can contaminate milk, human and animal bodies, medical devices, soil, water, and other environmental features. Thus, keen attention should be paid to the epidemiological surveillance and drug resistance of *Acinetobacter lwoffii* in the listed sources.

## Methods

### Sample collection and isolation of bacteria

In 2021, four raw milk samples were collected from cows diagnosed as subclinical mastitis on one farm in Jilin Province in China. The collection was performed aseptically, and the samples were placed in sterile tubes and immediately stored under refrigerated conditions until analysis.

In the laboratory, each milk sample was inoculated on Trypticase Soy Agar plates supplemented with 5% sheep blood and incubated aerobically at 37 °C for 48 h. After bacterial growth, colonies for suspicious pathogens were further sub-cultured for identification. For DNA extraction, 300 µL 1×TE buffer was added to a small proportion of the bacterial colony and transferred to a 1.5 mL centrifugation tube. The tube was hit at 100 °C for 10 min before incubation on ice for 5 min. The mixture was then centrifuged at 17,000 × g for 5 min, and the supernatants were collected and stored at 4 °C till further use. The isolates were identified using PCR by targeting 16s rRNA using universal primers. The quality of the PCR products was analyzed using 1% agarose gel electrophoresis and visualized under UV light. All PCR amplified positive products were sequenced by Kumi Biotechnology (Jilin) Co., Ltd (Jilin, China) and identified using the BLAST program using data in the National Center for Biotechnology Information (NCBI) database.

### Genetic evolutionary analysis

The bacterial sequences were aligned using the ClustalW program in MEGA 7.0 software. The phylogenetic trees from evolutionary distances were built using the neighbor-joining method. P-distances for nucleotides were reconstructed using the same software. The referential strains of *Acinetobacter* were all published strains. The clustering stability of the neighbor-joining tree was evaluated by bootstrap analysis with 1,000 replicates.

### Antimicrobial susceptibility testing

The antimicrobial susceptibility of the isolates was tested using the Kirby-Bauer disk diffusion antibiotic testing method (K-B method) through microdilution as recommended by the Clinical and Laboratory Standards Institute. The susceptibility and resistance tests of the isolates were performed against 23 different antibiotics, including ampicillin (AMP, 10 µg), oxacillin (OX, 1 µg), ceftazidime (CAZ, 30 µg), cefoxitin (CFO, 30 µg), cefazolin (CFZ, 30 µg), ceftriaxone (CRO, 30 µg), ceftiofur (TIO, 30 µg), erythromycin (ERY, 15 µg), ciprofloxacin (CIP, 5 µg), levofloxacin (LEV, 5 µg), enrofloxacin (ENR, 5 µg), clindamycin (CC, 2 µg), chloramphenicol (CLO, 30 µg), tetracycline (TET, 30 µg), doxycycline (DOX, 30 µg), polymyxin (CT, 30 µg), amikacin (AMK, 30 µg), streptomycin (STR, 10 µg), gentamicin (GEN, 10 µg), kanamycin (KAN, 30 µg), trimethoprim/sulfamethoxazole (SXT, 1.25/23.75 µg), imipenem (IPM, 10 µg) and meropenem (MPM, 10 µg). *Escherichia coli* ATCC 25,922 was the quality control strain.

The bacterial isolates were inoculated in Trypticase Soy Broth and incubated at 37 °C for 6 to 8 h until turbidity developed to 0.5 McFarland’s standard. A small bacterial culture inoculum was spread onto sterile Mueller Hinton agar plates using sterile cotton swabs. The plates were incubated at 37 °C for 16 h, and the diameters of growth inhibition zones were recorded.

### Detection of resistance genes

The bacterial colonies from an overnight culture were added to 300µL 1×TE buffer, boiled for 10 min, and cooled on ice for 5 min to release DNA. The PCR amplification of beta-lactamase genes (*bla*_*CMY−2*_, *bla*_*TEM*_, *bla*_*SHV*_, *bla*_*DHA*,_*and bla*_*CTX−M*_), aminoglycoside resistance genes (*aac(3’)-IIc, aac(3’)-IV, aph(2’)- Ib, aph(3’)-II, aph(3’)-IV, aph(3’)-VII, aadA1, aac(6’)-Ib, rmtA, rmtB, rmtC, rmtD, rmtE, armA, npmA*), chloramphenicol resistance genes (*cat1, cat2, cmlA, cmlB*), tetracycline resistance genes (*tet(A), tet(B), tet(C), tet(K), tet(M), tet(G)*), sulfonamides resistance genes (*sul1, sul2, sul3*) and plasmid mediates resistance genes (*qnrA, qnrB, qnrC, qnrD, qnrS, qepA, oqxAB*) were carried out using primers published article in a previous article [44]. All PCR products were sequenced, and alignments between nucleotides for the sequence data were performed using the BLAST tool to confirm the identity of the isolated bacteria.

## Data Availability

The datasets of the current study are available from the corresponding author upon reasonable request.
